# Reproductive Enhancement through Phytochemical Characteristics and Biological Activities of Date Palm Pollen: A Comprehensive Review on Potential Mechanism Pathways

**DOI:** 10.3390/metabo14030166

**Published:** 2024-03-14

**Authors:** Saad Salhi, Abdellatif Rahim, Mouad Chentouf, Hasnaa Harrak, Jean Loup Bister, Naima Hamidallah, Bouchra El Amiri

**Affiliations:** 1 Animal Production Unit, Regional Center for Agricultural Research of Settat, National Institute for Agricultural Research (INRA), Avenue Ennasr, P.O. Box 415, Rabat 10090, Morocco; 2 Laboratory of Biochemistry, Neurosciences, Natural Resources and Environment, Faculty of Sciences and Techniques, Hassan First University of Settat, P.O. Box 577, Settat 26000, Morocco; naima.hamidallah@uhp.ac.ma; 3 Animal Production Unit, Regional Center for Agricultural Research of Tangier, National Institute for Agricultural Research (INRA), Avenue Ennasr, P.O. Box 415, Rabat 10090, Morocco; 4 Plants Improvement and Quality Unit, Regional Center for Agricultural Research of Marrakech, National Institute for Agricultural Research (INRA), Avenue Ennasr, P.O. Box 415, Rabat 10090, Morocco; 5 Unit of Molecular Physiology, Faculty of Medicine, NARILIS, University of Namur, 5000 Namur, Belgium

**Keywords:** *Phoenix dactylifera* L., pollen, phytochemical composition, biological activity, reproduction parameters

## Abstract

Infertility represents a significant global health challenge affecting both men and women. Despite regular unprotected sexual intercourse, approximately 15% of couples of reproductive age struggle to conceive within 12 months, with 10% of infertility cases attributed to unknown causes worldwide. As a result, numerous studies have turned their attention to exploring the use of natural products for the prevention and treatment of infertility. Among these natural remedies is date palm pollen (DPP), a male reproductive powder derived from the blossoms of the *Phoenix dactylifera* L. palm tree, which has a long history of use as a dietary supplement, particularly as an aphrodisiac and fertility enhancer for both men and women. This review critically examines the diverse components of DPP, including metabolites, proteins, amino acids, fatty acids, to elucidate its potential impact on human reproduction. The analysis thoroughly assesses the composition of DPP in relation to its effects on enhancing reproductive processes and delves into its traditional uses and therapeutic benefits in male fertility, such as the enhancement of sexual desire, semen quality, and hormonal equilibrium. Similarly, it explores the influence of DPP on female fertility, emphasizing its potential to improve factors such as lubrication, desire, ovulation, and hormonal balance. Overall, this review underscores the potential of DPP as a natural remedy for addressing reproductive disorders.

## 1. Introduction

Infertility stands as a significant global health concern affecting both men and women. Approximately 15% of couples of reproductive age, despite regular unprotected sexual contact, face difficulties in conceiving within 12 months, with 10% of cases attributed to unknown factors [1]. Consequently, there has been a growing interest in exploring natural products for the prevention and treatment of infertility [2]. Furthermore, the prevalence of infertility in both developing and developed countries has prompted research into alternative approaches to address fertility issues. Within this framework, natural products such as date palm pollen (DPP) have garnered attention as potential interventions [3]. Extensive investigations into the effects of DPP on male and female reproductive parameters have been conducted to mitigate fertility-related problems. As a result, its notable ameliorative effects have been underscored in numerous clinical and subclinical studies [4,5,6]. Nevertheless, a comprehensive understanding of its impacts on male and female reproductive parameters is imperative for the effective utilization of the therapeutic potential of DPP.

The date palm (*Phoenix dactylifera* L.), a monocotyledonous plant belonging to the Arecaceae family, is primarily found in arid regions of the Middle East and North Africa [7]. Date palm pollen (DPP), extracted from the date palm, accounts for approximately 1000 tons of production annually, derived from millions of palm trees cultivated in Arabian regions. DPP represents the male reproductive cells of palm flowers and serves as a natural reservoir of various bioactive compounds, including estradiol, estriol, cholesterol, estrone, saponins, carbohydrates, fatty acids, tannins, and flavonoids [8,9,10,11]. These diverse compounds contribute to its considerable nutritional value and diverse therapeutic effects [9,12]. Moreover, DPP exhibits significant antioxidant and antimicrobial activities owing to the presence of volatile unsaturated fatty acids, phenolic compounds, carotenoids, and tocopherols [9,13].

This review aims to comprehensively analyze the correlation between the composition of DPP and its impacts on reproduction by conducting a thorough examination of the various secondary metabolites, proteins, amino acids, carbohydrates, fatty acids, minerals, and vitamins found in DPP. Additionally, it investigates the traditional uses of DPP and its therapeutic effects on male fertility, including its potential to enhance sexual desire, semen quality, and hormonal levels. Subsequently, it delves into its influence on female fertility, highlighting its potential to improve lubrication, desire, ovulation, as well as various biochemical and hormonal factors. This study extends to unraveling the possible mechanisms implicated in these effects on both male and female reproductive parameters. While existing reviews have explored the effects of date palm pollen on reproductive parameters, this review stands out by delving into potential mechanisms and the involvement of specific components of date palm pollen in shaping these reproductive outcomes.

## 2. Status of Date Palm in the World

The date palm (*Phoenix dactylifera* L.), a popular perennial fruit tree belonging to the Arecaceae family, holds considerable agricultural and economic importance globally [14]. Renowned for its ability to thrive in harsh desert conditions, the date palm has been cultivated in regions such as North Africa, the Arabian Peninsula, and the Middle East, where it plays a vital role as a representative species within desert ecosystems [15,16]. Over the past three centuries, its cultivation has expanded to various parts of the world, including Australia, India, Mexico, Pakistan, Southern Africa, South America, and the United States of America [7]. Presently, the global date palm population has exceeded 120 million trees, with the Middle East region alone contributing to 70% of this total [17]. Within this diverse palm population, 5000 different date varieties are cultivated worldwide. Notably, Morocco holds a significant share of this diversity, with the INRA cataloging a total of 453 Moroccan date palm varieties. Among these, the Mejhoul, Boufeggous, Bouskri, and Jihel varieties stand out prominently [18]. Additionally, internationally recognized date varieties include Ajwa, Zahidi, Aseel, Majdool, Mabrook, Dhakki, Halawi, Lasht, Deggla, and Bamy [19,20].

**Figure 1:**
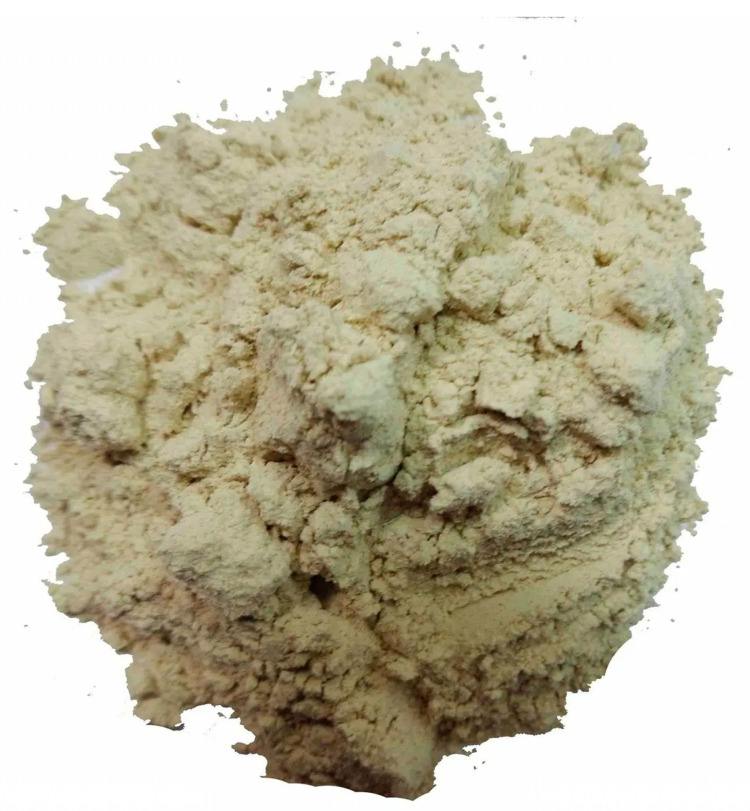
Date palm pollen powder.

Apart from its fruits, the date palm offers various secondary by-products such as leaves, coir, petioles, spadix stems, and trunks, serving multiple traditional purposes [21]. Originally cultivated for its date fruits, the date palm is renowned for its nutritional and medicinal properties owing to its richness in biomolecules [3]. It has been utilized to create value-added products like date flour, fiber concentrate, juices, jam, date fruit bars, sugar, and various dairy and bakery products, rendering the date palm an economically valuable commodity [22]. In the sphere of date palm pollen, only a few male date palm trees are needed to ensure the pollination process, with one male producing enough pollen to fertilize 50 female trees [23]. The remaining pollen is utilized in the Moroccan pharmacopeia to treat or prevent different diseases, mainly those related to reproduction in both males and females, further enhancing its economic value (Figure 1). It has attracted attention for its potential therapeutic effects, particularly in the realm of fertility. Studies suggest that DPP may positively impact reproductive health by enhancing sperm quality and count in males, as well as regulating menstrual cycles and improving ovulatory function in females [4,24,25]. Moreover, the historical application of date palm pollen as a revitalizing medicinal agent by early Egyptians and Chinese adds a rich layer to its narrative [10]. Previous studies delving into pollen’s impact on the reproductive system have further demonstrated its enhancing effects, encompassing anticoccidial, antiapoptotic, and antimicrobial properties [26,27].

## 3. Exploring Mechanisms: How Date Palm Pollen Composition Influences Reproduction?

The nutritional value of DPP has been recognized for an extensive period. Early Egyptians and ancient Chinese civilizations utilized DPP as a rejuvenating medicinal agent, often referring to it as the “fountain of youth”. Furthermore, owing to its nutritional content, DPP has traditionally served as an aphrodisiac and fertility enhancer [4]. Consequently, recent research has focused on the biochemical and nutritional characterization of this natural product. These studies collectively underscore the abundance of secondary metabolites in DPP, primarily antioxidants. The quantity of these metabolites varies depending on factors such as the DPP variety, growth soil, and climatic conditions [28,29,30]. Additionally, DPP represents a significant source of proteins, essential amino acids, carbohydrates, fatty acids, minerals, and vitamins [31].

### 3.1. Secondary Metabolites

DPP contains a diverse array of secondary metabolites (Table 1), including phenolic compounds [11], triterpenoids, sterols, and carotenoids. These organic compounds serve functions that extend beyond plant growth and reproduction, playing roles such as pest defense, pollinator attraction, and adaptation to environmental stress [32,33]. Consequently, these secondary metabolites possess a spectrum of bioactive properties that render them valuable for various human applications [34]. Certain secondary metabolites of DPP have been the subject of research for their potential effects on various aspects of reproduction, including fertility, hormonal balance, and reproductive health (Table 1).

#### 3.1.1. Phenolic Compounds

Considerable quantities of phenolic compounds, including caffeic acid, gallic acid, coumaric acid, catechin, chlorogenic acid, quercetin, and rutin, along with flavonoids such as isorhamnetin, apigenin, lutein, and naringin, have been identified in Egyptian, Tunisian, Moroccan, and Iraqi DPP [38,40,41] (Table 2). Additionally, other studies have highlighted pyrogallol and catechin as the major phenolic compounds present in DPP [9,42]. Throughout these investigations, substantial variability in terms of the concentration and types of phenolic compounds has been observed. This variability can be ascribed to various biological factors, including differences in genetics and cultivation practices, as well as environmental factors such as soil conditions, maturation stages, salinity levels, temperature, water availability, and light intensity [43]. Several experimental studies have examined the effects of phenolic compounds from DPP (administered at doses ranging from 100 to 360 mg/kg body weight (BW)/day) on sexual and reproductive functions in laboratory animals) [44,45,46,47]. All of these studies have reported that aqueous and ethanolic extracts of DPP enhance testicular health, hormone levels, and sexual behavior. Notably, the research has documented an improvement in mounting frequency, testosterone levels, sperm count, motility, and morphology, alongside histological and physiological changes in reproductive organs. Furthermore, the beneficial effects of these extracts on body weight and antioxidant status have also been observed. These studies offer valuable insights into the potential impacts of the phenolic compounds and flavonoids present in DPP on sexual parameters and reproduction [45,48,49]. However, it is important to note that these studies have not fully elucidated the precise mechanisms through which these biomolecules enhance reproductive characteristics. The positive results observed with both aqueous and ethanolic extracts of Date Palm Pollen (DPP) strongly suggest potent biological activity within its polar fraction. These extracts, each targeting distinct solubility profiles, reveal the presence of bioactive polar compounds like phenolic acids and flavonoids. These constituents, known for their affinity to cellular components such as enzymes, receptors, and signaling pathways crucial for reproduction, are often associated with robust biological effects. The combined effectiveness of both extracts implies a significant contribution of polar molecules, likely concentrated in these fractions, to the observed improvements in reproductive health parameters. Further research exploring the specific polar compounds present in DPP and their precise mechanisms of action is essential to elucidate their pivotal roles in promoting reproductive function.

**Figure 2:**
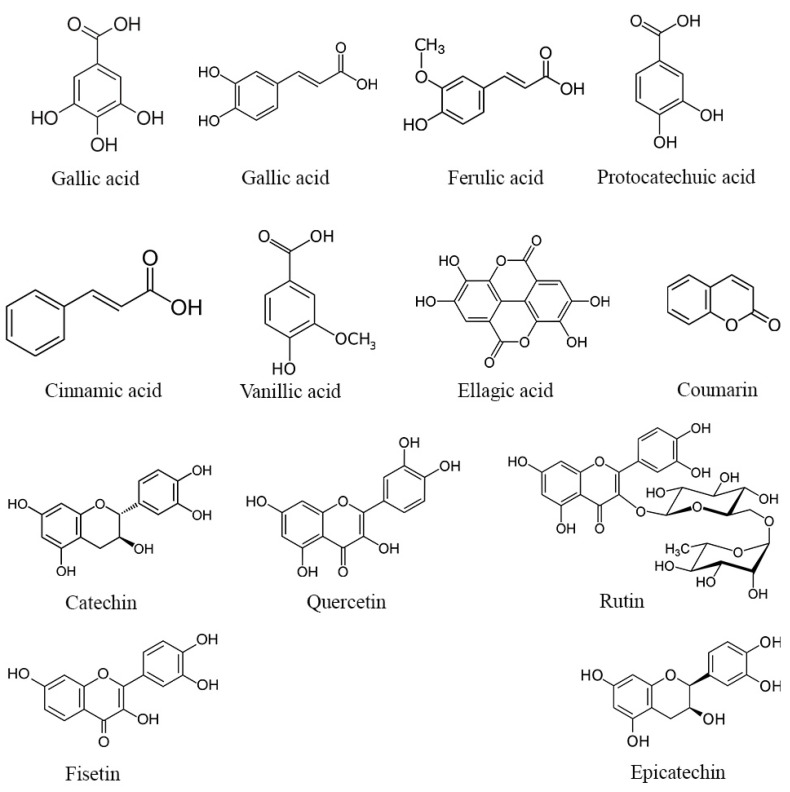
Structure of the main phenolic compounds found in DPP.

While the complete mechanisms underlying the potential enhancement of reproductive parameters by phenolic compounds and flavonoids in DPP remain incompletely understood, several hypotheses warrant consideration. It is plausible that these bioactive molecules might exert their effects through the modulation of hormonal pathways, including the potential influence on testosterone, luteinizing hormone, and estradiol levels, all of which play pivotal roles in reproductive function. For example, Jenkinson et al. [50] reported that phenolic compounds present in red wine can elevate testosterone levels by inhibiting its glucuronidation and subsequently reducing its urinary excretion. Furthermore, the antioxidant properties of phenolic compounds and flavonoids could potentially contribute to the reduction in oxidative stress within the reproductive system, thereby promoting healthier testicular architecture and function [51]. Several phenolic compounds possess both hydrophilic and lipophilic properties, rendering them amphiphilic in nature [52]. This unique property allows them to attach to cell membrane surfaces, potentially forming lipid bilayers and interacting with hydrophobic lipid chains. Through these interactions, polyphenols such as the gallic, α-coumaric, and ellagic acids (structures shown in Figure 2) can scavenge free radicals by H-atom transfer, resulting from lipid peroxidation, thereby safeguarding cell membranes and their contents from oxidative damage [53]. Moreover, it has been reported that these phenolic compounds enhance the antioxidant defense system by activating the extracellular signal-regulated kinase/nuclear transcription factor–erythroid 2-related factor 2 (ERK/Nrf2) pathways [54,55]. This activation leads to an upsurge in the expression of antioxidant enzymes, thereby contributing to the observed antioxidant activity displayed by certain phenolic compounds [55].

**Table 2:** Main phenolic compounds found in DPP.

Main Phenolic Compounds	Origin of DPP	References
Gallic acid, protocatechuic acid, chlorogenic acid, vanillic acid, caffeic acid, ferulic acid, alpha-coumaric acid, ellagic acid, cinnamic acid, salycilic acid, pyrogallol, catechin, catechol, epicatechein, caffeine, coumarin, reversetrol, narengin, hesperidin, rutin, quercetrin, rosmarinic, quercetin, naringenin, hesperitin, kaempferol, rhamnetin, apigenin, acacetin	Aswan governorate, northern Egypt	Ibrahim et al. [38]
Gallic acid, caffeic acid, ferulic acid, cinnamic acid, catechin, rutin, quercetin	Tata oasis region, south-east of Morocco	Salhi et al. [11]
Protocatechuic acid, vanillic acid, ellagic acid, rutin, quercetin, fisetin	Kerkennah and Tozeur regions, east and south-east of Tunisia	Daoud et al. [43]
Gallic acid, vanillic acid, caffeic acid, catechin, epicatechein, coumarin, rutin, quercetin	Biskra, south-east of Algeria	Benouamane et al. [56]
Chlorogenic acid, caffeic acid, cinnamic acid, catechin, rutin, quercetin, kaempferol, apigenin	Sharkia governorate, northern Egypt	Abdallah et al. [57]
Gallic acid, protocatechuic acid, chlorogenic acid, vanillic acid, caffeic acid, ferulic acid, alpha-coumaric acid, benzoic acid, ellagic acid, cinnamic acid, salycilic acid, pyrogallol, catechin, catechol, epicatechein, caffeine, coumarin, reversetrol, narengin, hesperidin, rutin, quercetrin, quercetin, hesperitin, kaempferol, rhamnetin, apigenin, acacetin	Riyadh, central Saudi Arabia	Abou Zeid et al. [58]
Gallic acid, chlorogenic acid, caffeic acid, catechin, rutin, quercetin	Alexandria, Egypt	El-Kholy et al. [9]

#### 3.1.2. Hormones

As early as 1947, Hassan and El Wafa [59], reported that the water-soluble form of the non-saponified fraction of the oil extracted from DPP exhibited similar cornification of the vaginal smear in rats, comparable to those injected with pure estradiol dipropionate. Consequently, these authors suggested the presence of biologically estrogenic substances within DPP. Previous investigations have also unveiled the presence of estrogenic materials in DPP extracts. Indeed, gonadotropic molecules derived from DPP were initially extracted by Soliman and Soliman [60], who highlighted the presence of a suitable Follicle-stimulating hormone (FSH) and luteinizing hormone (LH) -like in pollen grain extracts, inducing reproductive actions in rats. Subsequently, similar studies were carried out by El-Ridi et al. [61] and, more recently, by Otify et al. [62]. Additionally, Mahran et al. [63] isolated a steroidal saponin glycoside and a glycoprotein demonstrating gonadotrophic activity. They also confirmed the presence of estrone through thin-layer chromatography. However, to date, no study has succeeded in fully purifying and identifying gonadotropic hormones from DPP.

### 3.2. Proteins and Amino Acids

The protein content of DPP has exhibited a substantial range, varying from 15.81% to 38.18% [35,64] (Table 3). This considerable variability in protein content across studies can primarily be attributed to factors such as genotype, geographic origin, soil type, and climate conditions under which date palms are cultivated. For instance, Bacha et al. [64] investigated protein contents of 13 cultivars of date palms grown in Riyadh, Saudi Arabia, revealing protein contents ranging from 15.81% to 18.02%. Similarly, in Iraq, a study focusing on DPP of two cultivars demonstrated that the Ghannamy Ahmar pollen displayed a higher crude protein content of 27.24% compared to the Samasmi pollen, which contained 23.42% [39]. Furthermore, various studies conducted in Giza, Egypt, on the protein content in DPP of the El Hayani variety showed contents ranging between 30% and 33%, highlighting consistent protein richness [9,10,24,65]. Conversely, an investigation carried out in the Sohag governorate on the same variety demonstrated an even higher protein content, reaching 36% [66]. Similar results were obtained in Moroccan DPP, which contains about 35% of crude proteins [11]. The highest protein content was recorded in Deglet Nour DPP collected from Sfax, Tunisia, with 38.18% [35]. Despite the variations among studies, the protein content in DPP remains notably higher compared to date fruit, which does not exceed 6.5% [67,68]. DPP has also been described as a rich source of essential amino acids, particularly arginine (0.15–5.17%), valine (1.81–5.16%), histidine (1.61–2.53%), isoleucine (1.49–4.37%), leucine (3.43–8.35%), lysine (2.95–7.73%), methionine (0.11–2.37%), phenylalanine (1.63–4.25%), and threonine (1.72–4.70%) [10,35].

**Figure 3:**
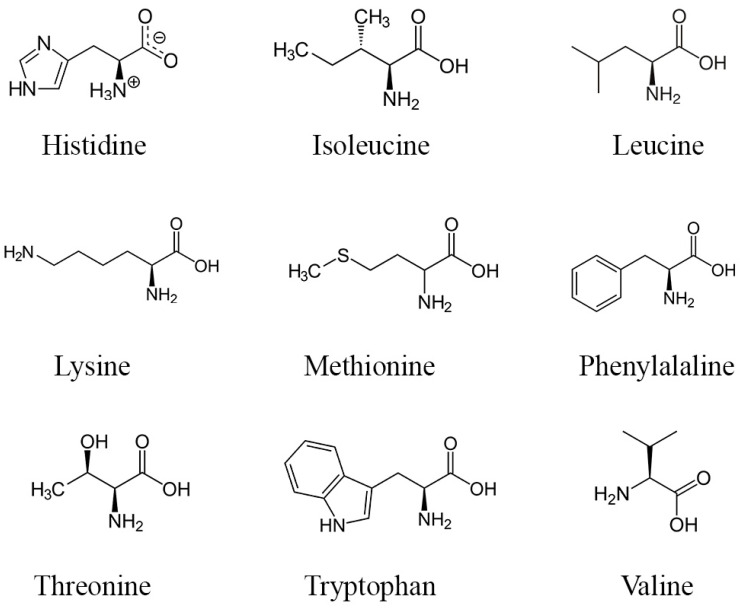
Structure of the main essential amino acids found in DPP.

Proteins and amino acids present in DPP may potentially impact fertility, yet further research is necessary to delineate their precise mechanisms as current knowledge remains incomplete. Nevertheless, amino acids and proteins found in DPP might enhance fertility through various mechanisms. Amino acids serve as the fundamental constituents of proteins, crucial for the synthesis of sexual hormones, facilitating the conversion of precursor molecules into active hormones via specialized proteins known as enzymes [69]. Sexual hormones, such as testosterone and estrogen, typically bind to carrier proteins for stable transportation to target tissues [70]. Upon reaching these target tissues, these hormones interact with specific receptor proteins on cells, instigating gene expression alterations and eliciting cellular responses by initiating reactions [71]. Hormone production is regulated by feedback mechanisms involving proteins to maintain a delicate balance [72]. Moreover, research has indicated that specific amino acids, including arginine (molecular structures in Figure 3), can enhance sperm quality and function by reducing heat and oxidative stress without adverse effects [73,74,75].

**Table 3:** Amino acid composition of DPP.

Amino Acids	3 Letter Code	1 Letter Code	DPP	DPP	DPP	DPP	DPP
Alanine	ala	A	2.14–8.36	2.61	6.48	2.71	2.43–8.27
Arginine	arg	R	1.18–1.73	1.61	5.77	1.42	1.18–1.74
Aspartic acid	asp	D	4.48–3.13	3.55	10.41	1.53	3.23–4.70
Cysteine	cys	C	N/A	0.42	1.11	0.91	N/A
Glutamine	gln	Q	2.58–4.66	1.74	13.23	2.23	3.91–4.71
Glycine	gly	G	8.46–7.81	2.24	5	1.81	4.40–8.19
Histidine	his	H	1.14–1.64	1.61	2.53	1.89	0.82–1.84
Isoleucine	ile	I	2.38–1.54	1.49	4.37	1.51	1.39–2.57
Leucine	leu	L	3.65–2.21	3.34	8.35	3.51	1.99–3.72
Lysine	lys	K	1.92–3.21	2.95	7.73	2.81	2.87–3.77
Methionine	met	M	0.41–6.64	0.11	2.37	0.14	0.50–0.80
Phenylalaline	phe	F	1.19–1.87	1.63	4.25	1.73	1.15–1.90
Proline	pro	P	1.53–2.04	0.28	N/A	N/A	1.73–2.28
Serine	ser	S	0–1.88	1.89	5.74	0.45	0.72–1.92
Threonine	thr	T	0.55–1.46	1.72	4.7	1.66	0.62–1.91
Tryptophan	trp	W	N/A	N/A	N/A	N/A	N/A
Tyrosine	tyr	Y	0.01–0.16	1.55	3.46	0.82	0.37–1.53
Valine	val	V	2.77–1.88	1.81	5.16	1.92	1.24–2.80
References			Bishr and Desoukey [13]	H. M. Hassan [10]	Sebii et al. [35]	Basuny et al. [36]	Aly [76]

### 3.3. Carbohydrates and Fatty Acids

Several studies have examined the carbohydrate content in DPP across different varieties and regions (Table 4). The highest percentage was observed in Moroccan DPP at 26.51% [11], followed by the Ghannamy Ahmar variety grown in Iraq, which reached a percentage of 26.25% [37]. Another study reported that Iraqi Khikri and Samsami DPP contain 16.27% and 22.78% of carbohydrates, respectively [39]. Additional research conducted on the El-Hayani variety cultivated in Egypt reported that carbohydrate levels vary between 13.41% and 17.10% [9,10,24]. Regarding fatty acids, a study carried out in Egypt on DPP from the El-Hayani variety identified 13 fatty acids, with palmitic acid (34.45%) and linoleic acid (14.24%) having the highest concentrations [10]. Additionally, oleic, linolenic, stearic, margaric, behenic, arachidonic, and lignoceric acids were also detected at high levels in Egyptian and Moroccan DPP [9,11,77]. Furthermore, palmitic, oleic, and linoleic acids were the major fatty acids identified in the Khikri and Samsami varieties of Iraqi DPP [39].

**Figure 4:**
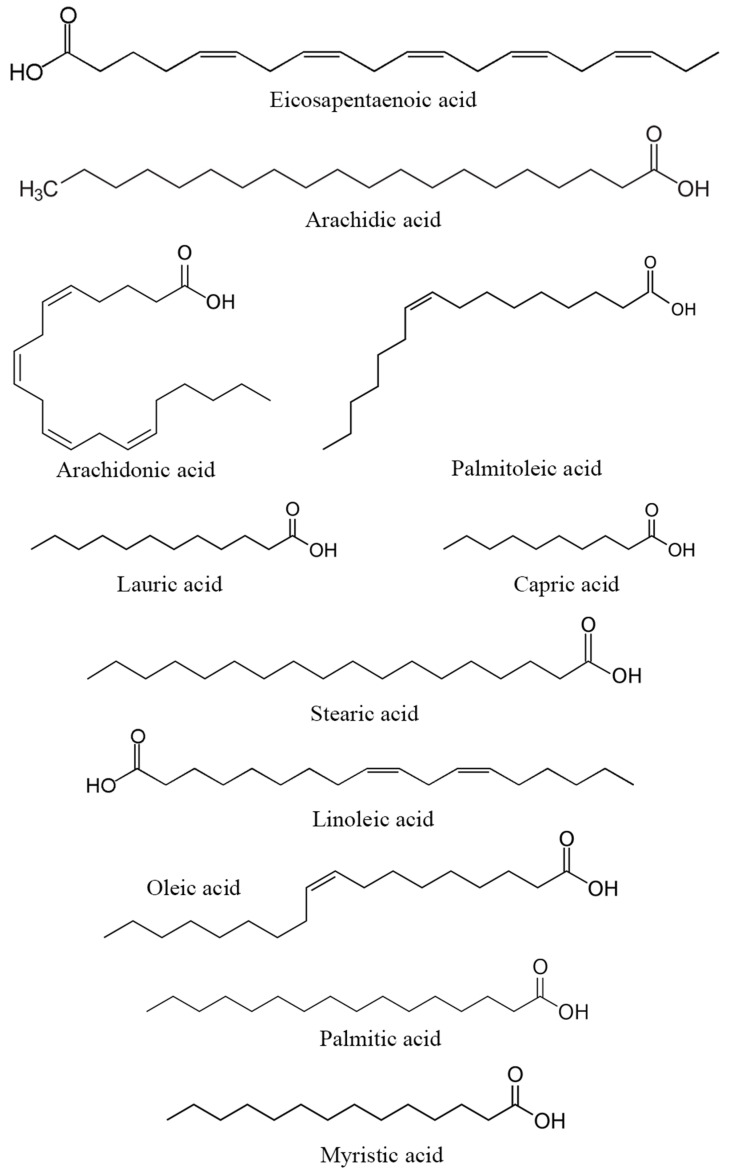
Structure of the main fatty acids found in DPP.

Similar to proteins and amino acids, the specific contribution of carbohydrates and fatty acids from DPP to reproduction has not been extensively investigated. However, these compounds constitute a part of the nutritional content of DPP and could potentially indirectly influence reproductive processes. Carbohydrates serve as an energy source for reproductive tissues through the bonds between carbon and hydrogen atoms (fatty acid structure highlighted in Figure 4). [78], while fatty acids play a crucial role in the structure and function of cell membranes [79]. Essential fatty acids, including omega-3 and omega-6, participate in physiological processes that may potentially impact reproduction. Certain types of fatty acids contribute to the synthesis and regulation of hormones, including prostaglandins, which play a role in vital processes such as ovulation and implantation. While their roles are plausible, the scientific studies directly linking DPP carbohydrates and fatty acids to human reproductive processes are currently limited.

### 3.4. Minerals and Vitamins

The DPP of the Egyptian variety El Hayani was found to contain significant amounts of minerals, including zinc (125–309 mg/100 g), calcium (60.5–560 mg/100 g), potassium (160–750 mg/100 g), selenium (305 mg/100 g), magnesium (130–318 mg/100 g), iron (241–226 mg/100 g), molybdenum (302 mg/100 g), copper (319.6 mg/100 g), manganese (170–310 mg/100g), and cobalt (305 mg/100g) [9,10,24,65,66]. However, variations observed between these studies could be attributed to factors such as the collection site and specific soil conditions at each location, as noted above for other compounds [80]. Moreover, DPP from the El-Ghannmi Ahmar cultivated in Iraq has demonstrated even higher mineral contents compared to Egyptian DPP. Specifically, the Iraqi DPP revealed elevated levels of potassium (7350 mg/100 g), magnesium (1960 mg/100 g), calcium (1080 mg/100 g), iron (8500 mg/100 g), and copper (365 mg/100 g), in comparison to Egyptian DPP [37] (Table 5).

Regarding vitamin content, a study focused on the Egyptian El-Hayani DPP indicated the presence of substantial levels of several vitamins, including high amounts of vitamins A (7708.33 IU/100 g), E (3030.92 IU/100 g), and C (89.09 mg/10 g). Another Egyptian study, involving four DPP varieties treated similarly and collected from comparable soil and climate conditions during the same period Bishr and Yehia, [13] revealed high amounts of vitamins B2 (260 mg/g) and B12 (2316 mg/g) in El Hayani, followed by Zaghlol (B2: 193 mg/g; B12: 182 mg/g), Amhat (B2: 15 mg/g; B12: 43 mg/g), and Sewy (B2: 10 mg/g; B12: 14 mg/g). Additionally, the Ahmat variety contained the highest amount of vitamin B1 (60 mg/g), followed by Sewy (44 mg/g), El Hayani (13 mg/g), and Zaghlol (11 mg/g) (Table 6).

**Figure 5:**
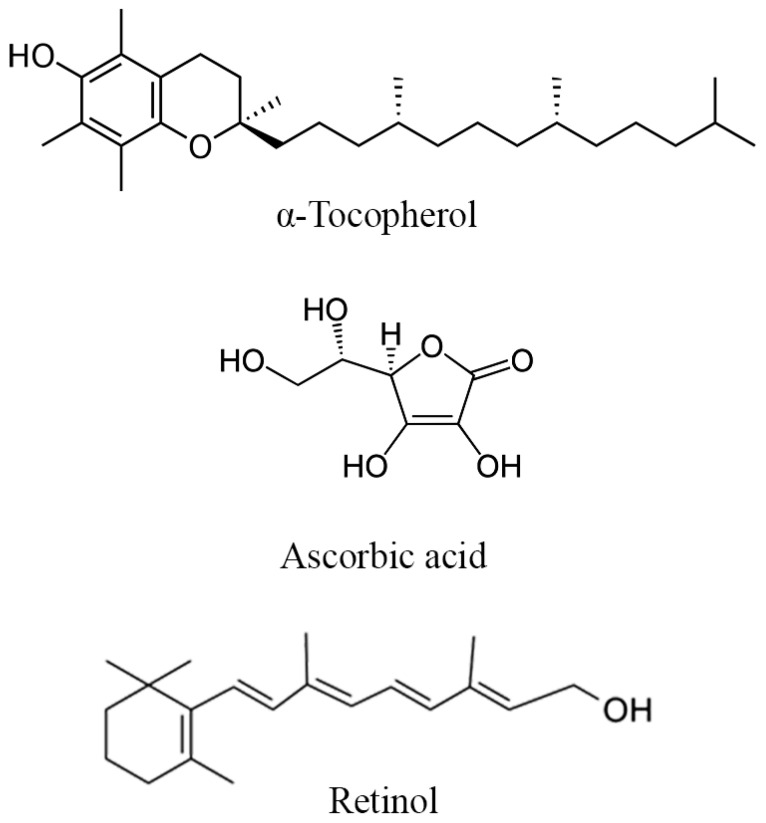
Structure of the main vitamins found in DPP.

The potential mechanism behind the plausible improvement of reproductive parameters by minerals and vitamins in DPP could be related to their roles in cellular function, antioxidant defense, and optimization of hormonal balance. For instance, zinc is necessary for the production and secretion of testosterone from the Leydig cells [81], enhancing desire and general sexual health, ovulation, and fertilization [82]. Calcium and magnesium levels influence the smooth muscle spasms that occur during ejaculation, potentially enhancing sexual function [83,84,85]. Iron contributes to oxygen transport and energy metabolism [86], factors that can indirectly impact reproductive functions. Antioxidant vitamins like A, E, and C are particularly crucial in counteracting oxidative stress through the release of electrons to free radicals (Structure of vitamins in DPP highlighted in Figure 5), which can have detrimental effects on sperm quality and reproductive organs [87,88,89]. By neutralizing free radicals, these vitamins can protect sperm from DNA damage, improve motility, and enhance overall fertility [90].

## 4. Effect of Date Palm Pollen on Human Reproduction

### 4.1. Traditional Utilization of Date Palm Pollen to Enhance Human Fertility

In historical contexts, pollen formulations have been widely distributed globally, serving as food additives and supplements to enhance overall dietary consumption [65,91]. Additionally, small quantities of DPP are selectively harvested for direct human consumption. For instance, pollen candies, a combination of pollen with honey or molasses and chocolate, have been marketed as a healthful food product in the United States of America [92]. Furthermore, reports indicate that both pollen-infused candy and capsules containing pollen collected from bees’ hind legs as they return to their hives are linked to numerous health benefits [92]. This practice finds proponents notably in Sweden and England [93]. Moreover, early Egyptians and ancient Chinese recognized the rejuvenating properties of DPP, often referred to as the “fountain of youth”, using this botanical substance as a therapeutic treatment for revitalization [10]. The investigation of various date palm varieties with medicinal properties from 17 oases in southern Algeria has revealed that pollen from Ghardaïa, Biskra, and Adrar is commonly employed in traditional medicine to address male and female fertility issues [94]. Traditional practices suggest combining this pollen with bee honey for both genders. In women, a popular method involves consuming a mixture of pollen powder and honey daily before breakfast, particularly during the ovulation period. Another technique includes blending pollen with herbal extracts and applying it to a sanitary towel during the fertile phase to promote ovulation and uterine health. This approach is believed to assist with uterine cleansing, lubrication, and fertility [94]. The use of DPP to enhance human fertility is also widespread in Morocco and Tunisia [95]. In these countries, it is consumed directly or in combination with pure honey and/or royal jelly. This convergence of traditional indigenous knowledge and modern studies underscores the significance of DPP in the realm of human reproduction.

In response to global fertility challenges, clinical studies are increasingly dedicated to examining the effects of DPP on human fertility [49,96,97]. These studies aim to evaluate how this product might influence fertility and reproductive health. While the current research may not primarily focus on deciphering the specific mechanisms, their exploration contributes to a deeper understanding of the potential impact of DPP on human fertility. By merging traditional knowledge and scientific research, these studies offer hope of providing valuable insights that could aid in addressing significant fertility issues faced by numerous individuals worldwide.

### 4.2. Therapeutic Effects of Date Palm Pollen on Male Fertility: In Vivo Assay

#### 4.2.1. Male Sexual Desire Disorders

**Figure 6:**
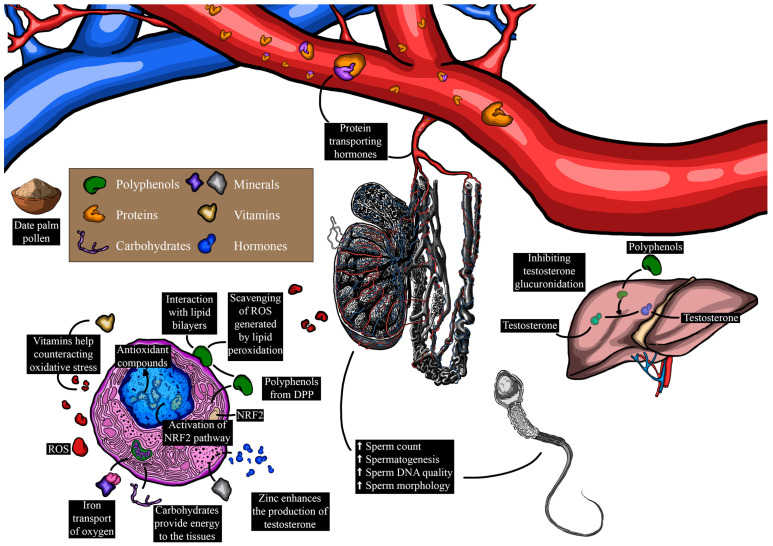
Influence of date palm pollen components on male reproduction and the underlying mechanisms (ROS: reactive species; Nrf2: nuclear factor erythroid 2-related factor 2).

Infertility, affecting approximately 15% of couples, represents a pressing challenge with broad societal and health implications [1]. Male infertility is a complex issue that impacts a substantial number of individuals, with often elusive causes [98]. This intricate matter encompasses various factors, including physiological aspects such as secondary hypogonadism [99], genetic factors like Y chromosome microdeletions [46,100], behavioral contributors such as smoking and medication use [101], environmental influences like exposure to toxic substances [102], and socio-demographic variables including age and profession [103]. Despite the prevalence and complexity of male infertility, the field has lacked empirically recommended, evidence-based pharmaceutical interventions, leading to a reliance on traditional pharmaceutical approaches assessed through clinical trials. Within this context, the potential therapeutic efficacy of DPP has emerged as a promising avenue. Table 7 and Table 8 and Figure 6 summarize the primary pharmacological effects of DPP on male reproductive parameters. Male sexuality is widely recognized as a complex mechanism, and sexual dysfunction represents a highly prevalent issue. It is characterized by disturbances in sexual behavior and sensation, encompassing problems such as erectile dysfunction, difficulties with intercourse, and diminished libido [104]. Various physiological systems, including the nervous, cardiovascular, endocrine, and reproductive systems, contribute to maintaining normal sexual function [105,106,107]. Disruption of these systems or psychosocial aspects can lead to sexual dysfunction [108]. Epidemiological research has established connections between male sexual dysfunction and various disorders, notably type II diabetes [109] and cardiovascular diseases [110]. In the realm of reproductive medicine, limited therapies have prompted researchers to explore natural products as potential treatments [111,112,113]. Of these, DPP has garnered considerable attention. It has been traditionally used as an aphrodisiac for patients with sexual dysfunctions [5], and recent clinical studies have delved into its pharmacological effects on enhancing sexual function. In this vein, Al-Sanafi et al. [114] reported that the co-administration of 500 mg of pollen powder and 100 mg of zinc sulfate capsules twice daily for 3 months promoted sexual desire in infertile men. Similarly, Marbeen et al. [115] found that the consumption of 500 mg of DPP powder twice daily for 3 months yielded similar results in infertile men. Furthermore, a double-blind controlled clinical trial demonstrated that the daily administration of 300 mg of DPP powder capsule over 30 days improved male sexual function [97]. Specifically, this dose of DPP significantly enhanced erectile function, orgasmic function, sexual desire, intercourse satisfaction, and overall satisfaction [97]. In a recent double-blind controlled clinical trial conducted on Iranian men after coronary artery bypass graft (CABG) with sexual dysfunctions, the consumption of 3 g of DPP powder twice a day for two months significantly increased their International Index of Erectile Function (IIEF) and Hurlbert Index of Sexual Desire (HISD) scores (from 23.21 to 46.57 and from 59.39 to 64.45, respectively) over time [116]. This study suggested that DPP has a cardiotonic effect due to its polyphenol contents, primarily involved in vasodilation through nitric oxide production [116], which translates into penile erection [117]. Moreover, the beneficial effects of DPP on sexual function and behavior may also be attributed to its androgenic properties [41]. Androgens, including testosterone, play a crucial role in regulating the magnitude of the penile erectile response, venous outflow from cavernous spaces, and sexual desire [101,118]. Additionally, DPP contains significant amounts of steroids, flavonoids, saponins, and lipids, which stimulate endogenous testosterone levels by raising the level of LH [119]. DPP also contains alkaloids that play a crucial role in inhibiting erectile dysfunction-related enzymes (arginase and PDE-5), leading to penile erection [120].

#### 4.2.2. Sperm Quality and Hormonal Levels

Semen quality stands as a major predictor of male fertility outcome [121]. Consequently, male infertility may arise from the gradual decline in sperm quality, influenced by a variety of factors, spanning from genetic mutations to lifestyle choices, medical illnesses, or medications [122]. Rasekh et al. [123] investigated the effects of the same dosage administered to a group of 40 infertile men through gelatinous capsules every two days instead of daily, revealing a significant increase in sperm count, motility, and morphology after two months of treatment. Marbeen et al. [115] also noted a substantial rise in sperm count and active sperm motility in infertile patients treated with 500 mg of DPP twice daily for 3 months. Similarly, Al-Sanafi et al. [114] reported that the coadministration of the same dose of DPP (500 mg) and 100 mg of zinc sulfate capsules twice daily for 3 months increased serum LH, FSH, and testosterone levels, while improving sperm count and active sperm motility. In an Iranian case report study involving a man with idiopathic severe oligoasthenoteratozoospermia, normal morphology, total motility, progressive motility, and sperm concentration impressively increased [124]. The results from these studies illustrate the ameliorative effects of DPP not only on semen quality parameters but also on the reproductive system as a whole. These beneficial effects have been attributed to the abundance of phenolic compounds in DPP [3,41], known for their antioxidant activities. In this vein, a recent study by Falahati et al. [47] indicated that the consumption of 400 mg/kg of body weight over 74 days in infertile men significantly improved semen volume, count, and morphology by reducing reactive oxygen species (ROS) and increasing the expression of antioxidant genes, such as peroxiredoxin-1 (PRDX1) and peroxiredoxin-6 (PRDX6). This finding may support the notion that phenolic compounds present in DPP could induce the expression of nuclear factor-erythroid factor 2 (Nrf2) [125,126,127], a transcription factor regulating the expression of peroxiredoxin antioxidant genes [128]. Furthermore, a controlled clinical trial demonstrated that the administration of 400 mg/kg of gelatinous capsules daily for 30 days by infertile men increased the expressions of Nrf2, glutathione peroxidase (GPx4), superoxide dismutase (SOD2), and catalase (CAT) genes [129]. The study emphasized that this increase in antioxidant genes was positively correlated with semen quality [129].

Despite the low oxygen tensions characterizing the testicular microenvironment, the testicular tissue remains vulnerable to oxidative stress. Consequently, improved serum levels of testosterone rely on enhanced testicular antioxidant activity, achieved through the activation of the testicular endocrine and antioxidant systems [130]. The testes heavily depend on major reactive oxygen species (ROS) processing enzymes, as well as small molecular weight antioxidant factors, including minerals such as zinc and copper, and vitamins C and E. DPP, abundant in these biomolecules, provides zinc and copper, recognized as cofactors for free radical scavenging enzymes like superoxide dismutase (SOD), and protectors of sulfhydryl groups. Zinc is also known to impede lipid peroxidation by displacing transition metals like iron and copper from catalytic sites. Vitamin E, a potent lipophilic antioxidant, is crucial for maintaining mammalian spermatogenesis [131]. It is found in particularly high concentrations in Sertoli cells and pachytene spermatocytes, and to a lesser extent in round spermatids [132]. Vitamin C (ascorbic acid) contributes to supporting spermatogenesis, at least partially through its ability to reduce α-tocopherol and maintain this antioxidant in an active state. Vitamin C is itself kept in a reduced state by a GSH-dependent dehydroascorbate reductase, which is abundant in the testes [133]. Deficiencies in vitamins C or E result in a state of oxidative stress in the testes, disrupting both spermatogenesis and testosterone production [131]. Vitamin E has also been found to suppress lipid peroxidation in testicular microsomes and mitochondria [122,125]. DPP, rich in vitamin C and E, as well as caffeic acid, which has been shown to decrease MDA value and increase GPx activity in the testes, could explain its beneficial effect on the antioxidant system, leading to improved testosterone synthesis. In a recent study, Karimi et al. [49] reported that a daily dose of 6 g of DPP dry powder in two separate doses (3 g every 12 h) administered orally to 30 eligible men for three months significantly increased serum testosterone levels (from 5.31 ± 0.40 ng/mL to 6.88 ± 0.71 ng/mL). Steroidal components also play a vital role in regulating the renewal of spermatogenic cells and male reproductive tissues that possess estrogen receptors [126,127]. Moreover, it has been reported that saponins present in DPP stimulate Leydig cells, consequently increasing testosterone production [3,128], a crucial component in spermatogenesis [134].

**Table 7:** Main in vivo experimental studies highlighting potential effects of date palm pollen (DPP) on different aspects of reproductive parameters *.

Test Group	Form of DPP	Type of Administration	Doses of DPP	Time	Results	Reference
Male rats	Aqueous extract	Intraperitoneal injection	140 and 350 mg/kg BW twice a day	120 min	↑ Mount, intromission and ejaculation frequency; ↑ ejaculation latency; ↑ post-ejaculatory interval; ↑ index of libido; ↑ blood levels of testosterone and estradiol; ↑ penile erection; ↓ mount and intromission latency	Abedi et al. (2012) [44]
			140 mg/kg BW/day	70 min	↑ Mount and intromission frequency; ↑ ejaculation latency; ↑ release of dopamine levels; ↑ penile erection; ↓ ejaculation frequency; ↓ mount and intromission latency	Abedi et al. (2014) [119]
	Aqueous extract	Oral administration	100 mg/kg BW/day	4 weeks	↑ Testicular histological architecture and integrity; ↑ testis weight; ↑ serum testosterone, LH and FSH levels; ↑ sperm count, motility and viability; ↑ sexual desire; ↓ testicular nitric oxide levels; ↓ malondialdehyde levels	Mohamed et al. (2018) [45]
	Ethanolic extract		150 mg/kg	56 days	↑ Body, epididymis, prostate gland, seminal vesicle and testis weight; ↑ serum LH, testosterone and estradiol levels; ↑ testicular antioxidant status; ↑ sperm count and motility; ↑ DNA integrity; ↓ pro-apoptotic markers expression; ↓ DNA damage	El-Kashlan et al. (2015) [46]
	Aqueous extract		120 mg/kg BW/day	35 days	↑ Serum testosterone levels; ↑ body weight; ↑ Johansen score	Iftikhar et al. (2011) [100]
					↑ Body and testis weight; ↑ serum and intratesticular testosterone levels	Yasir et al. (2014) [111]
	Aqueous extract		120, 240 and 360 mg/kg BW/day	35 days	↑ Sperm count and motility; ↑ seminiferous tubules diameter; ↑ germinal cell layer thickness; ↑ Leydig and spermatogonia cells; ↑ serum testosterone, LH and estradiol levels; immotile sperm; ↓ sperm abnormality	(Mehraban et al. (2014) [48]
Female mice	Aqueous extract	n.d.	100, 200, 400 mg/kg BW	n.d.	↑ Testosterone, estrogen and progesterone levels; ↑ number of antral and secondary follicles	Hosseini et al. (2014) [135]
Female rats	DPP water suspension	Oral administration	150 mg/kg BW/day	6 weeks	↑ Serum FSH and LH levels	Hammed et al. (2012) [136]
	Ethanolic extract	Oral administration	100 mg/kg BW/day	28 days	↑ GSH, FSH and LH levels	Jiheel and Arrak (2015) [137]

* BW: body weight; LH: luteinizing hormone; FSH: follicle-stimulating hormone; GSH: glutathione reduced; ↑: increase; ↓: decrease; n.d: not determined.

**Table 8:** Pharmacological effects oral administration of date palm pollen (DPP) powder on different aspects of men reproductive parameters *.

Test Group	Doses of DPP	Time	Results	Reference
Infertile men	500 mg twice daily	3 months	↑ Sexual desire; ↑ sperm count; ↑ active sperm motility; ↑ serum testosterone, LH and FSH levels	Al-sanafi et al. (2023) [114]
	500 mg twice daily	3 months	↑ Sexual desire; ↑ sperm count; ↑ active sperm motility; ↑ serum testosterone, LH and FSH levels; ↑ intercourse rate	Marbeen et al. (2005) [115]
	400 mg/kg BW	74 days	↑ Semen volume, count and morphology.	Falahati et al. (2023) [47]
	120 mg/kg BW every 2 days	2 months	↑ Sperm count, motility and morphology	Rasekh et al. (2015) [123]
	300 mg per day	30 days	↑ Erectile function; ↑ orgasm sexual desire; ↑ intercourse satisfaction	Jahromi et al. (2022) [97]
Men	3g twice a day	2 months	↑ Vasodilatation; ↑ IIEF; ↑ HISD	Hooshang et al. (2022) [116]
	3 g every 12 h	3 months	↑ Serum testosterone	Karimi et al. (2023) [49]
Man: case report	3 g every 12 h	3 months	↑ Sperm morphology, total and progressive motility and concentration	Karimi et al. (2018) [124]

* BW: body weight; LH: luteinizing hormone; FSH: follicle-stimulating hormone; IIEF: International Index of Erectile Function; HISD: Hurlbert Index of Sexual Desire; ↑: increase; ↓: decrease.

### 4.3. Therapeutic Effects of Date Palm Pollen on Female Fertility: In Vivo Assays

Despite the limited number of studies evaluating the effect of DPP on female reproduction, the year 2017 marked the initiation of several clinical trials (Table 9). Sadeghi et al. [138] observed that the administration of DPP capsules (350 mg daily) for 35 days improved vaginal lubrication and reduced dyspareunia in postmenopausal women compared to the control group receiving a starch placebo. Additionally, another trial demonstrated that 300 mg of DPP supplementation for 35 days in non-menopausal women improved the lubrication and desire domains of the Female Sexual Function Index, without significantly affecting arousal, orgasm, satisfaction, and pain compared to the control group receiving 300 mg of the starch capsule [113]. Another study reported that the oral administration of the same dose increased arousal, orgasm, lubrication, pain during intercourse, and satisfaction in women [97]. Moreover, Yosefzadeh et al. [112] showed that this dose improved orgasm in postmenopausal women without affecting sexual satisfaction. However, Loripoor et al. [25] reported that the daily administration of 300 mg of DPP aqueous extract for 4 weeks did not significantly reduce the score of sexual dysfunctions in postmenopausal women. In all these studies, the same dose of pollen was used for the same duration; however, variations in the date palm genotypes used in each study and the dissimilar composition of pollen from various date palms, particularly in terms of amino acids [139], may contribute to the differences in these results. Moreover, the results obtained by Loripoor et al. [25] may also be explained by the possible absence of bioactive compounds in the aqueous extract of DPP that are implicated in the improvement of sexual dysfunction. El-Wahed et al. [96] also demonstrated that daily consumption of 3 g of DPP for 3 months improved sex hormone levels in women with polycystic ovarian syndrome, leading to lower estrogen and LH levels, higher progesterone and FSH levels, and a cumulative impact on ovulation.

**Figure 7:**
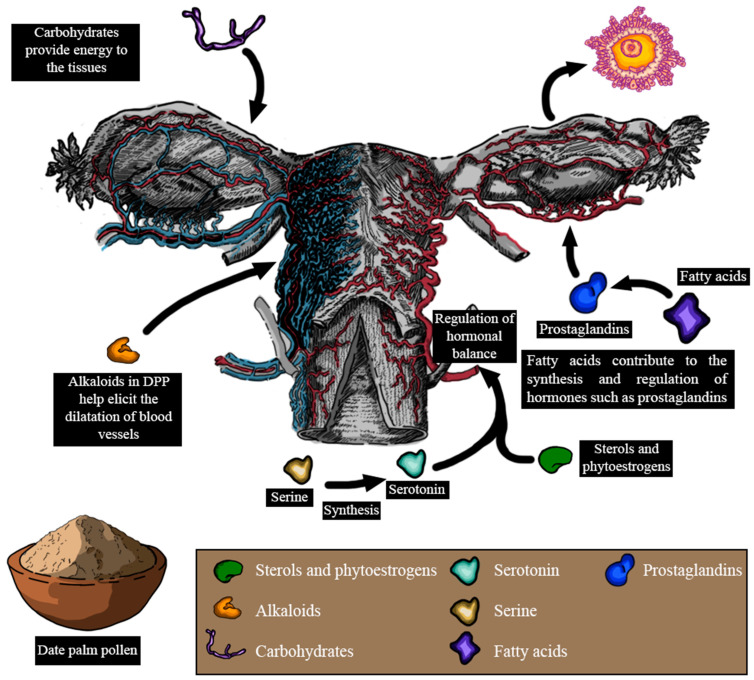
Pharmacological effects of date palm pollen on female reproduction and the plausible mechanisms implicated in some of these effects (LH: luteinizing hormone; FSH: follicle-stimulating hormone).

According to these studies, it has become evident that DPP could improve lubrication, desire domains, sexual hormone levels, and ovulation, as well as reduce dyspareunia (Figure 7). However, the mechanisms through which DPP elicits these effects remain unclear. The enhancement in lubrication and desire found after DPP supplementation might be due to several factors. DPP contains sterols and phytoestrogens, which have been suggested to regulate hormonal balance and potentially influence sexual desire [96]. Moreover, the vitamins and amino acids found within DPP could indirectly support libido by promoting overall health and boosting energy levels. L-arginine is a naturally occurring amino acid that plays a pivotal role in circulation and sexual function [140]. It serves as a precursor to nitric oxide (NO) and acts as a crucial mediator in this process. Through the action of nitric oxide synthase (NOS), L-arginine is converted into NO, increasing its level and cyclic guanosine monophosphate (cGMP). This biochemical cascade ultimately influences circulation and sexual function [141]. Serine, another amino acid found in DPP, could be involved in the synthesis of serotonin, a neurotransmitter known to play a pivotal role in regulating sexual desire [142]. Additionally, the carbohydrates present in DPP could provide a quick energy boost, potentially enhancing interest in sexual activities. Gauthaman and Ganesan reported that alkaloids present in DPP possess ergogenic properties that are capable of eliciting the dilation of blood vessels. Hence, by promoting vasodilation, DPP could potentially contribute to improving vaginal blood circulation, leading to enhanced lubrication and comfort [143]. In addition, psychological variables and interactions with the vaginal flora, as well as DPP’s historical usage as an aphrodisiac, might all play a role.

### 4.4. Regulatory Imperatives, Toxicological Effects, and Research Directions for Date Palm Pollen in Fertility Enhancement

Despite the promising therapeutic potential of DPP in addressing human fertility concerns, its broader application necessitates thorough toxicological assessments to identify potential adverse effects and establish safe dose thresholds. In rats and mice, it was reported that the dose of DPP affects reproduction parameters. For instance, Abedi et al. [44] indicated that 140 mg/kg of aqueous DPP extract improved rat sexual behavior more effectively than 350 mg/kg, with the latter causing adverse effects like diarrhea. Additionally, Mehraban et al. [48] observed the dose-dependent effects of oral DPP administration (120, 240, and 360 mg/kg) on reproductive parameters in rats. While the 120 and 240 mg/kg doses showed significant enhancements in reproductive metrics, the higher dose of 360 mg/kg did not yield substantial differences compared to controls. However, in human, the majority of studies have focused on assessing the impact of DPP on fertility using a single dosage [45,46,100,111]. Therefore, comprehensive studies elucidating its toxicological profile, including acute and chronic toxicity assessments, mutagenicity, reproductive toxicity, and potential interactions with medications, remain critical gaps in current knowledge. The establishment of regulatory frameworks is imperative, encompassing standardized production methods, quality control measures, and robust labeling standards to ensure product safety, efficacy, and proper consumer information. Future research avenues should emphasize long-term safety evaluations across diverse populations, exploring mechanistic insights into DPP’s effects on reproductive systems. Large-scale clinical trials and epidemiological studies are essential to validate its efficacy and safety, considering variations in demographic profiles and potential susceptibilities. Moreover, conducting comprehensive toxicological profiling will aid in delineating the exact mechanisms of action, facilitating the development of informed guidelines for its judicious use in addressing fertility concerns.

## 5. Conclusions

DPP presents a versatile natural resource abundant in secondary metabolites, proteins, amino acids, carbohydrates, fatty acids, minerals, and vitamins. Its capacity to positively impact reproductive health is promising, as its bioactive components may influence hormonal pathways and enhance fertility. The traditional use of DPP to enhance human fertility, combined with recent clinical research, emphasizes its potential therapeutic effects on both male and female reproductive parameters. DPP shows promise in improving sexual function, hormone levels, and semen quality. While variations in study outcomes do exist, the historical significance of DPP and ongoing research offer valuable insights into addressing fertility issues and promoting reproductive health. This comprehensive understanding underscores the importance of further exploration and application of DPP’s potential in the realm of human reproduction.

## Figures and Tables

**Table 1:** Approximate composition of DPP.

Parameter	DPP	DPP	DPP	DPP	DPP	DPP	DPP	DPP
Moisture (%)	28.8	N/A	30.31	29	8.041	8.14	44.88	9.59
Ash (%)	4.57	10.23	6.16	6.2	5.585	4.99	N/A	8.59
Crude fiber (%)	1.37	8.09	N/A	2.3	0.113	N/A	N/A	N/A
Crude fat (%)	20.74	10.8	10.24	31.5	7.678	N/A	8.5	N/A
Crude protein (%)	31.11	36.28	38.18	39.8	19.45	N/A	26.93	N/A
Carbohydrate (%)	13.41	17.14	18.22	20.2	26.25	3.78	22.78	N/A
Aw	N/A	N/A	0.898	N/A	N/A	N/A	N/A	0.45
pH	N/A	N/A	6.31	N/A	N/A	4.75	N/A	6.23
Reference	H. M. Hassan, [10]	El-Kholy et al. [9]	Sebii et al. [35]	Basuny et al. [36]	Al-Samarai et al. [37]	Ibrahim et al. [38]	Alanber [39]	Salhi et al. [11]

**Table 4:** Fatty acid composition of DPP.

Fatty Acids		DPP (µg/g)	DPP (IU/g)	DPP (IU/g)
Capric acid	(C10:0)	0.46	0.46	N/A
Lauric acid	(C12:0)	4.82	5.08	5.37
Myristic acid	(C14:0)	13.33	16.22	16.01
Palmitic acid	(C16:0)	34.45	24.24	34.63
Stearic acid	(C18:0)	2.04	3.43	3.9
Arachidic acid	(C20:0)	7.32	6.64	7.5
Palmitoleic acid	(C16:1n-7)	7.07	7.23	7.79
Oleic acid	(C18:1n-9)	7.19	7.11	7.45
Linoleic acid	(C18:2)	14.24	20.26	15.5
Arachidonic acid	(C20:4n-6)	4.57	0.57	N/A
Eicosapentaenoic acid	(C20:5n-3)	0.52	N/A	N/A
References		H. M. Hassan [10]	El-Kholy et al. [9]	Basuny et al. [36]

**Table 5:** Mineral and elemental composition of DPP.

Element	1 Letter Code	DPP	DPP	DPP	DPP	DPP	DPP
Carbon	C	26.80–30.16	N/A	N/A	N/A	N/A	N/A
Nitrogen	N	53.10–55.4	N/A	N/A	N/A	0.31	N/A
Oxygen	O	15.57–16.67	N/A	N/A	N/A	N/A	N/A
Magnesium	Mg	0–0.16	N/A	0.32	N/A	1.96	1.86
Phosphorous	P	0.31–0.93	N/A	N/A	0.39	N/A	N/A
Sulfur	S	0–0.69	N/A	N/A	N/A	N/A	N/A
Potassium	K	0–15.16	N/A	0.75	0.40	7.35	13.39
Calcium	Ca	0.20–26.38	N/A	0.56	0.21	1.08	4.92
Zinc	Zn	0–0.38	0.28	0.12	0.27	0.28	0.61
Iron	Fe	0–0.47	0.24	0.23	0.26	0.85	0.96
Manganese	Mn	0–0.33	0.28	0.07	0.30	0.27	0.52
Sodium	Na	0.06–0.22	N/A	N/A	N/A	0.43	0.19
Boron	B	N/A	0.31	N/A	N/A	0.30	N/A
Selenium	Se	N/A	0.30	N/A	0.32	0.25	N/A
Molybdenum	Mo	N/A	0.30	N/A	N/A	0.32	N/A
Copper	Cu	N/A	0.32	N/A	0.25	0.36	N/A
Cobalt	Co	N/A	0.30	N/A	N/A	0.20	N/A
Nickel	Ni	N/A	0.30	N/A	N/A	0.17	N/A
Cadmium	Cd	N/A	N/A	N/A	N/A	0.01	N/A
References		Bishr and Desoukey [13]	H. M. Hassan [10]	El-Kholy et al. [9]	Basuny et al. [36]	Al-Samarai et al. [37]	Ibrahim et al. [38]

**Table 6:** Vitamin composition of DPP.

Full Name	Vitamin	DPP (µg/g)	DPP (IU/g)	DPP (IU/g)
Retinol	A	N/A	77.0833	75.70
Thiamin	B1	11–60	N/A	N/A
Riboflavin	B2	10–260	N/A	N/A
Cobalamin	B12	14–2316	N/A	N/A
Ascorbic acid	C	N/A	0.90	1.51
Alpha-tocopherol	E	N/A	30.31	35.11
References		H. M. Hassan [10]	El-Kholy et al. [9]	Basuny et al. [36]

**Table 9:** Pharmacological effects of the oral administration of DPP on different aspects of women reproductive parameters *.

Test Group	Form of DPP	Doses of DPP	Time	Results	Reference
Menopausal women	Powder	350 mg/day	35 days	↑ Vaginal lubrication; ↓ dyspareunia	Sadeghi et al. (2018) [138]
		300 mg/day	35 days	↑ Orgasm	Yosefzadeh et al. (2017) [112]
Women		300 mg/day	35 days	↑ Lubrication; ↑ desire domains of the Female Sexual Function Index	Salmani et al. (2022) [113]
Women with PCOS		3 g/day	3 months	↓ Estrogen and LH levels; ↑ progesterone and FSH levels; ↑ ovulation	El-Wahed et al. (2022) [96]
Postmenopausal women	Aqueous extract	300 mg	4 weeks	No change in sexual dysfunction	Loripoor et al. (2023) [25]

* PCOS: polycystic ovarian syndrome; LH: luteinizing hormone; FSH: follicle-stimulating hormone; ↑: increase; ↓: decrease.

## Data Availability

Not applicable.
